# Nicotinamide Adenine Dinucleotide-Dependent Flavin Oxidoreductase of *Mycoplasma hyopneumoniae* Functions as a Potential Novel Virulence Factor and Not Only as a Metabolic Enzyme

**DOI:** 10.3389/fmicb.2021.747421

**Published:** 2021-09-29

**Authors:** Xing Xie, Fei Hao, Rong Chen, Jingjing Wang, Yanna Wei, Jin Liu, Haiyan Wang, Zhenzhen Zhang, Yun Bai, Guoqing Shao, Qiyan Xiong, Zhixin Feng

**Affiliations:** ^1^Key Laboratory for Veterinary Bio-Product Engineering, Ministry of Agriculture and Rural Affairs, Institute of Veterinary Medicine, Jiangsu Academy of Agricultural Sciences, Nanjing, China; ^2^College of Veterinary Medicine, Nanjing Agricultural University, Nanjing, China; ^3^Hubei Key Laboratory of Animal Nutrition and Feed Science, Wuhan Polytechnic University, Wuhan, China

**Keywords:** *Mycoplasma hyopneumoniae* (Mhp), NADH-dependent flavin oxidoreductase, adhesion, pathogenic, virulence factor

## Abstract

Mycoplasma hyopneumoniae (Mhp) is the main pathogen that causes enzootic pneumonia, a disease that has a significant impact on the pig industry worldwide. The pathogenesis of enzootic pneumonia, especially possible virulence factors of Mhp, has still not been fully elucidated. The transcriptomic and proteomic analyses of different Mhp strains reported in the literature have revealed differences in virulence, and differences in RNA transcription levels between high- and low-virulence strains initially indicated that nicotinamide adenine dinucleotide (NADH)-dependent flavin oxidoreductase (NFOR) was related to Mhp pathogenicity. Prokaryotic expression and purification of the NFOR protein from Mhp were performed, a rabbit-derived polyclonal antibody against NFOR was prepared, and multiple sequence alignment and evolutionary analyses of Mhp NFOR were performed. For the first time, it was found that the NFOR protein was conserved among all Mhp strains, and NFOR was localized to the cell surface and could adhere to immortalized porcine bronchial epithelial cells (hTERT-PBECs). Adhesion to hTERT-PBECs could be specifically inhibited by an anti-NFOR polyclonal antibody, and the rates of adhesion to both high- and low-virulence strains, 168 and 168L, significantly decreased by more than 40%. Moreover, Mhp NFOR not only recognized and interacted with host fibronectin and plasminogen but also induced cellular oxidative stress and apoptosis in hTERT-PBECs. The release of lactate dehydrogenase by hTERT-PBECs incubated with Mhp NFOR was significantly positively correlated with the virulence of Mhp. Overall, in addition to being a metabolic enzyme related to oxidative stress, NFOR may also function as a potential novel virulence factor of Mhp, thus contributing to the pathogenesis of Mhp; these findings provide new ideas and theoretical support for studying the pathogenic mechanisms of other mycoplasmas.

## Introduction

As the simplest self-replicating organism, *Mycoplasma* has the smallest genome. It is speculated that *Mycoplasma* evolved from the degeneration of gram-positive bacteria and are most closely related to certain clostridia in phylogeny (Razin et al., 1998; Peterson and Fraser, 2001). Due to its limited biosynthesis and metabolism capabilities, *Mycoplasma* relies on infecting host cells to obtain the nutrients necessary to meet the demands of its parasitic life style (Razin et al., 1998); thus, they have developed mechanisms to invade and then survive in host cells. The highly variable surface protein group is responsible for the rapid changes in the main surface protein antigens, the invasion of nonphagocytic host cells, and the subsequent regulation of the host immune system (Much et al., 2002; Buim et al., 2011; Chopra-Dewasthaly et al., 2012; Hegde et al., 2014; Burki et al., 2015). These mechanisms contribute to the establishment of chronic infection and to the persistence of *Mycoplasma* in the host.

Many species of mycoplasmas are pathogens that cause various diseases in livestock and eventually lead to heavy economic losses (Hegde et al., 2015; Maes et al., 2018). Among them, *Mycoplasma hyopneumoniae* (Mhp), the pathogen that causes porcine endemic pneumonia, is a highly contagious and globally distributed porcine respiratory pathogen. Mhp usually destroys the epithelium along with the respiratory tract, making pigs vulnerable to secondary infections by other bacteria and viruses and ultimately causing significant economic losses to the pig industry worldwide (Maes et al., 2018). However, a basis for the immune pathogenesis of Mhp has not yet been fully elucidated. Some scholars have considered that the onset of porcine enzootic pneumonia depends on Mhp virulence factors, which enable the pathogen to evade host defense mechanisms, as well as on the production of molecules that participate in processes including cell–host adhesion, response to environmental stress in the host, and immune regulation (Leal Zimmer et al., 2020). Therefore, identifying novel virulence factors would be of great importance for illuminating the mechanism underlying Mhp pathogenesis.

Despite the presence of surface proteins, a variety of metabolic enzymes have been identified and described as critical determinants of pathogenicity in addition to their roles in basic metabolism (Pilo et al., 2005; Rohmer et al., 2011). Currently, it has been reported that mycoplasmas can utilize GAPDH, enolase, pyruvate dehydrogenase, dihydrolipoamide dehydrogenase, Cpn (Hsp) 60, peptidylprolyl isomerase, glutamyl aminopeptidase, elongation factor thermo unstable (Ef-Tu), cytotoxic nuclease, oligopeptide permease, and protein Mhp82 as putative proteins that also act as virulence factors (Henderson, 2014). The utilization of available substrates and the metabolic potential and growth rate of bacteria all play indispensable roles related to pathogenicity (Hegde et al., 2015). A previous transcriptomics study demonstrated that nicotinamide adenine dinucleotide (NADH)-dependent flavin oxidoreductase (NFOR) was overrepresented in Mhp pathogenic strain 7448 but not in nonpathogenic *Mycoplasma flocculare* (Siqueira et al., 2014). In addition, previous comparative proteomics reports demonstrated that there are significant differences in the expression of NFOR between high- and low-virulence Mhp strains (Reolon et al., 2014; Li et al., 2019). NFOR is an oxidoreductase, the largest class of enzymes, and functions to catalyze the oxidation of NADH to NAD^+^ by reducing molecular O_2_ to H_2_O or H_2_O_2_ at the same time (Liu et al., 2014). Some organisms, such as *Nomuraea rileyi*, express these kinds of enzymes, which play important roles in regulating cellular redox and osmotic pressure balance to maintain normal cell growth and development (Liu et al., 2014). Therefore, we hypothesized that Mhp NFOR, in addition to its effect on metabolic processes, may have a certain relationship with Mhp virulence.

As a novel candidate virulence factor discovered in this study, NFOR was located at the surface of Mhp, as determined by electron microscopy and flow cytometry analyses. NFOR could adhere to immortalized porcine bronchial epithelial cells (hTERT-PBECs) and recognize host fibronectin and plasminogen, with higher affinity for fibronectin. NFOR could also induce host cytotoxicity, oxidative stress damage, and apoptosis. Our findings support the notion that NFOR may be a potential novel virulence factor of Mhp, and these findings will provide new ideas and theoretical support for studying the pathogenic mechanisms of Mhp and other mycoplasmas.

## Materials and Methods

### *Mycoplasma hyopneumoniae* Strains and Growth Conditions

All five Mhp strains were thawed from frozen Mhp bacterial stocks and subcultured for three generations before use for subsequent analysis. The strains were cultured in modified Friis medium, designated KM2 cell-free medium, which was supplemented with 20% (v/v) pig serum (sterilized by irradiation after being harvested from a clean snatch-farrowed, porcine colostrum-deprived piglet, and stored in our lab), and cultivated in a humidified incubator at 37°C (Xiong et al., 2014). Mhp strain 168 was isolated and cultured from a pig, which showed the typical symptoms of mycoplasma pneumonia of swine (MPS), in China (Ho et al., 1980). This field strain was gradually attenuated through continuous passage until the 380th generation, generating the low-virulence strain 168L (Liu et al., 2013); the Mhp 168L strain used in this study was passage 353. Strain JS is a virulent strain that can induce typical characteristics of MPS with a lung lesion score of approximately 15, as previously mentioned (Xiong et al., 2014). Strain LH is a virulent clinical strain that was isolated in our lab (GenBank accession number: CP079799). Strain J (ATCC 25934) was passaged once from the ATCC stock to yield frozen stocks. The titers of the Mhp strains were quantified using the 50% color change unit (CCU_50_) assay (Leigh et al., 2008), which was modified from the CCU assay (Furr and Taylor-Robinson, 1993), and investigated by quantitative PCR.

### RNA Transcriptional Analysis

The five Mhp strains were cultured under the abovementioned culture conditions at 37°C for 48 h. Then, total RNA was extracted using a Total RNA Extraction Kit (Cat No. R6834, Omega Biotek, Guangzhou, China). HiScript^®^ II Q RT SuperMix for qPCR (+gDNA wiper) (Cat No. R223-01, Vazyme, Nanjing, China) was used to reverse transcribe at least 1 μg of total RNA in a 10-μL reaction volume before performing qRT-PCR with an ABI 7500 Real Time PCR System with the HiScript^®^ II One Step qRT-PCR SYBR^®^ Green Kit (Cat No. Q221-01, Vazyme, Nanjing, China). qRT-PCR was performed in triplicate using cDNA of the *NFOR* gene under specific conditions. The P46 gene of Mhp was selected as the internal control. The PCR primers used in the quantitative assays are listed in Table 1. The fold changes in mRNA expression of the *NFOR* genes of various Mhp strains that differed in virulence were determined using the 2^–ΔΔCT^ method as previously described (Xie et al., 2018).

**TABLE 1:** Primers used in this study.

**Primer name**	**Summary of functions or sequences (5′–3′)**
NFOR-F	GGAACGAAGAGGCGGTAAT
NFOR-R	CAGGCACAAATGCTGAAGA
Mhp P46-F	TCACTTGCGGCGGGTCTAT
Mhp P46-R	TTGCTTGTTCGGCCATTCC
	For real-time PCR analysis of *NFOR* gene expression at the transcriptional level
Mhp183-F	CCAGAACCAAATTCCTTCGCTG
Mhp183-R	ACTGGCTGAACTTCATCTGGGCTA
Mhp183-P	FAM-AGCAGATCTTAGTCAAAGTGCCCGTG-TAMRA
	For real-time PCR analysis of *Mhp*

### Multiple Sequence Alignment

Seventeen amino acid sequences of the NFOR protein from Mhp were retrieved from the National Center for Biotechnology Information and UniProt protein databases, and their homologies were analyzed. All the sequences were aligned with the CLUSTAL W program (Larkin et al., 2007). Phylogenetic inference was performed through Molecular Evolutionary Genetics Analysis version 10 (MEGA 10, Kumar et al., 2018) according to the neighbor-joining criterion. A total of 2,000 non-parametric bootstrap analyses were used to test the robustness of the hypothesis.

### Protein Expression and Purification

The Mhp *NFOR* gene (MHP168_RS01740) was synthesized by GenScript Biotech Corp. (Nanjing, China) and was expressed in the BL21(DE3) *Escherichia coli* strain through the pET32a vector. Before Mhp NFOR protein expression, NFOR was analyzed for signal peptide predictions with SignalP-5.0 Server^1^ (Almagro Armenteros et al., 2019). The protein expression and purification processes were performed as previously reported with some modifications (Chen et al., 2019; Montfort-Gardeazabal et al., 2021). Bacterial cells were grown in LB medium at 37°C until the OD600 reached approximately 0.7–0.8, and then, IPTG was added to the culture at a final concentration of 0.25 mM and incubated at 16°C to induce protein expression. The cells were collected by centrifugation and resuspended in buffer A containing 30 mM Tris-HCl (pH 8.0), 300 mM NaCl, and 20 mM imidazole before the cells were lysed by sonication and centrifuged at 100,000 × *g* for 30 min. Then, the soluble fraction was incubated with Ni Sepharose 6 FF resin (GE Healthcare, Shanghai, China) at 4°C for 1 h, and then, the Mhp NFOR protein was eluted in buffer A by adding 200 mM imidazole and concentrated using Centricons (Amicon, Merck Ltd., Beijing, China) ultrafiltration. Subsequently, the proteins were dialyzed in buffer B containing 30 mM Tris-HCl (pH 8.0) and 300 mM NaCl. High Affinity Ni-Charged Resin FF affinity columns (Cat No. L00666, GenScript, Nanjing, China) were used for further purification. The purified protein concentrations were determined with the BCA Protein Assay Kit (Cat No. P0012S, Beyotime, Shanghai, China), and the final concentrations were expressed as milligrams of protein per liter of bacterial solution. The proteins were then stored at −70°C.

### Preparation of Polyclonal Antibodies and Western Blotting Analysis

Polyclonal antibodies against Mhp NFOR were obtained by subcutaneously immunizing 1-month-old New Zealand white rabbits with 1.2 mg of recombinant protein (rNFOR) emulsified in Freund’s complete adjuvant for the first immunization (Cat No. F5881, Sigma-Aldrich, St Louis, MO, United States). Each rabbit was then immunized with 1.2 mg of rNFOR emulsified in Freund’s incomplete adjuvant (Cat No. F5506, Sigma-Aldrich, St Louis, MO, United States) twice at 2-week intervals. Booster immunization was performed once a week after three immunizations before sera were collected.

Subsequently, rNFOR was identified by Western blotting analysis, which was performed in three independent replicates. Approximately 15 μg of rNFOR was resolved by 10% SDS-PAGE before being transferred to nitrocellulose filter membranes (Cat No. 77010, Thermo Fisher Scientific, Waltham, MA, United States). The membranes were blocked with blocking buffer [5% nonfat milk in Tris-buffered saline (TBS) containing 0.5% Tween 20 (TBST)] and were incubated at 37°C for 2 h. Then, the membranes were incubated with anti-NFOR serum at a dilution of 1:400 (produced in our lab) in blocking buffer in a 37°C incubator for 1.5 h. After three washes with TBST, the membranes were incubated with secondary antibody (HRP-labeled goat anti-rabbit IgG (H+L) (Cat No. BA1056, Boster, Wuhan, China) at 1:5,000 in blocking buffer for another 2 h. The membranes were incubated with electrochemiluminescence substrate reagents (Cat No. 32109, Thermo Fisher Scientific, Waltham, MA, United States) after three washes with TBST before the membranes were finally observed with a ChemiDoc XRS+ system (Bio-Rad, Hercules, CA, United States).

### Enzymatic Activity Assays

The enzymatic activity of purified rNFOR was determined by calculating the oxidation of NADH to NAD^+^ at 25°C. In brief, 5 μg/mL rNFOR, 0.1 M potassium phosphate buffer (pH 7.5, supplemented with 1 mM dithiothreitol), 10 μM flavin mononucleotide (FMN) (Cat No. F107158, Aladdin, Shanghai, China) and 0.5 mM NADH (Cat No. N106933, Aladdin, Shanghai, China) were incubated in triplicate in this assay. The purified rNFOR was preincubated with FMN for 5 min before NADH was added to an enzyme activity reaction system with a total volume of 2 mL. The optical density (OD) was measured at 340 nm (OD340). The specific activity was calculated by the following equation:


$$\frac{U}{m\,g}=\frac{△\,O\,D}{t\times \varepsilon \times l\times m}\times V\times 10^{6}.$$

### Surface NFOR Expression Detection by Flow Cytometry

Flow cytometry analysis was used to test whether NFOR is located on the surface of Mhp strains and to investigate differences in NFOR surface distributions between the high-virulence strain 168 and the low-virulence strain 168L. Mhp strains 168 and 168L (titers of which were 1 × 10^8^ CCU/mL) were incubated with anti-rNFOR serum or with preimmune serum (negative control) at a 1:100 dilution in triplicate, as reported previously (Zhu et al., 2017; Yu et al., 2018). Fluorescein isothiocyanate (FITC)-conjugated anti-IgG (Cat No. BA1105, Boster, Wuhan, China) was then used to stain the Mhp strains, and a BD Accuri C6 flow cytometer was used to measure the fluorescence intensity.

### Surface NFOR Expression Detection by Immunoelectron Microscopy

*Mycoplasma hyopneumoniae* strains were cultured and grown for approximately 60 h until the mid-log phase, with the color changing from red to light yellow, before each Mhp strain was harvested by centrifugation at 10,000 × *g* at 10°C for 20 min. A total of 1 × 10^8^ CCU/mL bacterial suspension was washed three times with ice-cold 0.1 M phosphate-buffered saline (PBS, pH 7.4) and finally resuspended in a volume of 50 μL of PBS. Immunoelectron microscopy was performed in triplicate according to protocols described in previous studies (Hegermann et al., 2008; Chen et al., 2019) with some modifications. Briefly, 5 μL of the sample was added to a 400 mesh formvar-coated nickel grid and incubated for 5 min. Then, the grid was fixed with 2% paraformaldehyde in PBS for 5 min at room temperature (RT) followed by blocking with 1% negative rabbit serum and blocking buffer [1% (w/v) BSA in PBS] for 1 h. The samples were then incubated with anti-rNFOR antibody or preimmune serum (negative control) at a 1:10 dilution in blocking buffer (PBS supplemented with 1% negative rabbit serum and 1% BSA) for another 1 h at RT (PBS was used as a blank control). After washing five times in blocking buffer, the samples were incubated with a secondary gold-conjugated antibody (goat anti-rabbit IgG, 10 nm-gold particles, Cat No. GA1014, Boster, Wuhan, China) at a dilution of 1:20 for 1 more hour. The samples were washed five times with PBS, for 5 min each. Subsequently, the samples were fixed with 2% paraformaldehyde in PBS for 5 min. Then, the grids were washed eight times with distilled water and stained with 1% phosphotungstic acid (pH 6.5) for 15 s. After the samples were dried by an infrared lamp, they were observed under a Tecnai high-field transmission electron microscope.

### Indirect Immunofluorescence Assay

Immortalized porcine bronchial epithelial cells were established and cultured to 80% confluence in 24-well cell plates with Dulbecco’s modified Eagle’s medium:nutrient mixture F-12 (DMEM/F12) medium plus 2% (v/v) fetal bovine serum (Gibco, Grand Island, NY, United States) supplemented with growth factors (Cat No. CC-4175, Lonza, Basel, Switzerland) as we previously reported (Xie et al., 2018). The cells in each group were seeded in three independent wells and washed three times with cold PBS before being fixed with 4% paraformaldehyde for 10 min at RT. Subsequently, 0.2% Triton X-100 was added and incubated at RT for 3 min, followed by blocking for 2 h using 3% (w/v) BSA in PBS. The cells were incubated with 100 μg of purified rNFOR for 1 h at 37°C in a cell incubator before they were washed three times with PBS and incubated with an anti-rNFOR antibody at a 1:250 dilution for another 2 h at 37°C. After three washes with PBS, the cells were then incubated with a 1:100 dilution of tetraethyl rhodamine isothiocyanate (TRITC)-conjugated anti-IgG (Cat No. SA00007-2, Proteintech, Rosemont, IL, United States) for 1 h in a 37°C incubator. 6-Diamidino-2-phenylindole (DAPI, Cat No. D8417, Sigma-Aldrich, St Louis, MO, United States) was used for nuclear staining before the cells were observed using a fluorescence microscope (Zeiss, Tokyo, Japan). Instead of rNFOR, BSA was selected as a negative control.

### Antibody-Mediated Adhesion Inhibition

*Mycoplasma hyopneumoniae* strains (the high-virulence 168 and low-virulence 168L strains, the titers of which were 1 × 10^7^ CCU/mL) were collected by centrifugation at 10,000 × *g* for 20 min at 10°C and resuspended in 500 μL of PBS after washing three times with PBS. The samples were prepared in three independent replicates and were preincubated with a polyclonal antibody against rNFOR or preimmune serum at a 1:20 dilution for 30 min in a 37°C incubator. Mhp bacteria were suspended in DMEM/F12 medium at 1 × 10^7^ CCU/mL, and one mL of bacteria were added per well to confluent hTERT-PBECs seeded in 24-well cell plates (3 × 10^5^ cells per well). The plates were then centrifuged at 1,000 × *g* for 10 min before being incubated at 4°C for 2 h. After washing three times with PBS, the hTERT-PBECs were collected after digestion with 0.125% trypsin (twice diluted with Hanks medium with 0.25% trypsin, Cat No. 25200072, Gibco, Grand Island, NY, United States), and the cells were centrifuged at 1,300 rpm for 10 min after adding DMEM/F12 containing 10% FBS to stop the cell digestion. After Mhp bacterial DNA extraction, quantitative real-time PCR was then performed as previously reported (Wu et al., 2012); the real-time PCR primers are shown in Table 1. The experiments were performed in triplicate, and the data were analyzed using SPSS 20.0. The Mhp titers were quantified using the CCU_50_ assay mentioned above.

### Surface Plasmon Resonance Analysis

Surface plasmon resonance (SPR) analysis was performed in triplicate according to the method described in our previous study (Chen et al., 2019) using a Biacore X100 Plus instrument (GE Healthcare, Boston, MA, United States). Fibronectin and plasminogen were diluted to 50 μg/mL before they were covalently linked to a CM5 sensor chip using an amine coupling kit (Biacore AB, Cytiva, Guangzhou, China). The immobilization of soluble fibronectin and plasminogen produced approximately 2,000 resonance units (RUs). The binding kinetics were measured by increasing the concentration (0–4,000 nmol/L) of the analyte (Mhp NFOR) in running buffer (HBS-EP), which consisted of 10 mM HEPES, 150 mM NaCl, 3 mM EDTA, and 0.05% (v/v) surfactant P20 (Biacore AB, Cytiva, Guangzhou, China), with a flow rate of 30 μL/min, passing through immobilized Mhp NFOR at 20°C for 3 min. The dissociation phase was monitored for 1,000 s by allowing buffer to flow through the chip. Biacore X100 control software was used to manually analyze the binding kinetics.

### Far-Western Blotting Analysis

Fifteen micrograms of rNFOR was resolved by 10% SDS-PAGE and then transferred to a PVDF membrane (Cat No. IPFL00010, Millipore, Darmstadt, Germany). After three washes with PBS, the membrane was blocked with 5% skim milk in TBST (TBS containing 0.5% Tween 20), which served as the blocking buffer, before being incubated in a 37°C incubator for 2 h with gentle shaking. Then, the membrane was incubated with 15 μg/mL fibronectin (Cat No. F1056, Sigma-Aldrich, Darmstadt, Germany) or plasminogen (Cat No. SRP6518, Sigma-Aldrich, Darmstadt, Germany) at 37°C for another 2 h. After another three washes with TBST, the membranes were subsequently incubated with an anti-fibronectin antibody (Cat No. ab299, Abcam, Cambridge, United Kingdom) at a 1:1,000 dilution or an anti-plasminogen antibody (Cat No. ABP55618, Annkine, Wuhan, China) at a 1:400 dilution in blocking buffer for 2 h in a 37°C incubator. The membranes were then incubated with a secondary antibody (HRP-conjugated goat anti-rabbit IgG) (Cat No. BA1055, Boster, Wuhan, China) at a 1:2000 dilution for 2 h in a 37°C incubator after three washes with TBST. Finally, the membranes were developed with Electro-Chemi-Luminescence (ECL) substrate using a ChemiDoc XRS+ system (Bio-Rad, Hercules, CA, United States). Instead of rNFOR, BSA was used as a negative control. The results of the assay were derived from three independent experiments and were consistent among the experiments.

### Quantification of Lactate Dehydrogenase Release

Immortalized porcine bronchial epithelial cells were seeded in 12-well cell plates at 80% confluence the night before the experiment. First, the cells were incubated with purified rNFOR protein at different concentrations (5, 10, 15, and 20 μg). Six hours later, the culture supernatants were collected, and lactate dehydrogenase (LDH) activity was measured with a CytoTox 96^®^ Non-Radioactive Cytotoxicity Assay (Cat No. G1780, Promega, Madison, WI, United States) following the manufacturer’s instructions. The corrected values in the formula below were used to calculate the percentage of cytotoxicity: percent cytotoxicity = 100 × experimental LDH release (OD_490_)/maximum LDH release (OD_490_). PBS was used instead of rNFOR as a negative control, and three Mhp strains (strains JS, J, and 168L, 1 × 10^8^ CCU/mL), which differed in virulence, were used as positive controls. Cells incubated with four concentrations of rNFOR, three strains of Mhp, or PBS were seeded in three independent wells and analyzed in three independent replicates.

### Reactive Oxygen Species Detection

Immortalized porcine bronchial epithelial cells were seeded in a 96-well plate at 80% confluence the night before the experiment. The cells were incubated with 20 μg of purified rNFOR, 80 μL of three strains of Mhp diluted in a final volume of DMEM/F12 plus 2% FBS culture medium, or 80 μL PBS. Then, 20 μL of H_2_O_2_ Substrate solution from the ROS-Glo^TM^ H_2_O_2_ Assay kit (Cat No. G8820, Promega, Madison, WI, United States) was added to the cells and mixed. Thus, the final well volume was 100 μL, and the final H_2_O_2_ Substrate concentration was 25 μM. The plate was incubated in a 37°C incubator for 6 h prior to adding 100 μL of ROS-Glo Detection Solution and incubated at RT for 20 min. The relative luminescence units (RLUs) were recorded using a plate reader. PBS was used as a negative control, and positive controls were the RLUs from three Mhp strains (strain JS, J, and 168 L, 1 × 10^8^ CCU/mL). Detection of reactive oxygen species (ROS) levels in the cells incubated with rNFOR, three Mhp strains or PBS was performed in five independent replicates.

### Apoptosis Assay

Immortalized porcine bronchial epithelial cells were seeded at a density of 2 × 10^5^ cells/well in a total volume of 500 μL culture medium in 24-well cell plates one night before the experiment. hTERT-PBECs were then incubated with 20 μg of purified rNFOR at 37°C for 12 h. Cells cultured in DMEM/F12 plus 2% FBS culture medium supplemented with epithelial growth factors F12 without the reagents mentioned above were used as a negative control, and cells incubated with Mhp strains that differed in virulence (the high-virulence strain JS and low-virulence strain 168L, 1 × 10^8^ CCU/mL) were used as positive controls. To explore whether antiserum specific for rNFOR could block and reduce the apoptosis induced by Mhp in hTERT-PBECs, we preincubated Mhp strains (JS and 168L strains, 1 × 10^8^ CCU/mL) with rabbit polyclonal antibodies raised against rNFOR at a 1:20 dilution at 37°C for half an hour before they were added to hTERT-PBECs seeded in 24-well plates and incubated in a 37°C incubator for 12 h. Three independent wells were prepared for each experimental group and the PBS negative control group, and three independent repeated trials were performed. The apoptosis rate was detected and calculated using a dual apoptosis detection kit (Cat No. A211, Vazyme, Nanjing, China) with Annexin V-FITC/PI.

### Statistical Analysis

All the experiments were repeated and carried out in triplicate, with the exception of ROS detection, which was performed in five independent replicates. Normality was investigated with the D’Agostino and Pearson omnibus normality tests. We used Student’s *t*-test to determine statistical significance between for paired samples. The data of the NFOR protein adhesion rate from quantitative real-time PCR analysis and the apoptosis rate were analyzed by GraphPad Prism 6 software and FlowJo software v7.6. Relative NFOR Differences in the mRNA expression levels between the moderate-virulence strain J or the low-virulence strain 168L and the high-virulence Mhp strains 168, JS, and LH were assessed via one-way analysis of variance (ANOVA) using SPSS Statics v20.0 software and GraphPad Prism 6 software. The adhesion rates between anti-NFOR serum or negative serum and groups of the high-virulence strain 168 as well as the adhesion rates between the low-virulence strain 168L and anti-NFOR serum or negative serum were compared by Student’s *t*-test. *P* < 0.05 was considered a significant difference, and *P* < 0.01 was considered an extremely significant difference.

## Results

### Significant Differences in NFOR Transcription Levels Between High- and Low-Virulence *Mycoplasma hyopneumoniae* Strains

**FIGURE 1:**
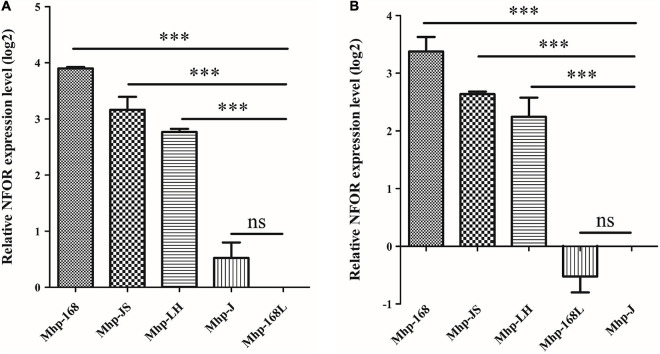
Relative expression levels of the NFOR genes from various Mhp strains that differed in virulence compared to the low-virulence Mhp strain 168L **(A)** and the moderate-virulence Mhp strain J **(B)** (set to 1). The relative NFOR gene expression level was confirmed by quantitative real-time PCR. The error bars represent standard deviations (SD) from three independent replicates (^∗∗∗^ indicates extremely significant difference with *p* < 0.001, ns indicates no significant difference with *p* > 0.05).

The relative quantitative RT-PCR results showed that there were indeed significant differences in the NFOR transcription levels among the five Mhp strains that differed in virulence. Figure 1 shows the mRNA expression levels of the *NFOR* gene in five Mhp strains, and the Mhp strains 168L (Figure 1A) and J (Figure 1B) were used as controls. The mRNA expression levels of the *NFOR* gene were significantly upregulated in the high-virulence Mhp strains 168, LH and JS compared to the low-virulence Mhp strain 168L. The mRNA expression levels of the *NFOR* gene in Mhp strain J remained unchanged, as the change in its expression levels were far lower than a 2-fold change (Figure 1A), which is usually considered the criterion for determining whether expression is significantly different (Xie et al., 2021). Similarly, the mRNA expression levels of the *NFOR* gene were significantly upregulated in the high-virulence Mhp strains 168, LH, and JS compared to the moderate-virulence Mhp strain J (Figure 1B). In contrast, the mRNA expression levels of the *NFOR* gene in Mhp strain 168L were decreased compared with those in strain J, but the difference was not significant.

### Bioinformatics Analysis, Protein Expression, and Enzymatic Activity of *Mycoplasma hyopneumoniae* NFOR

**FIGURE 2:**
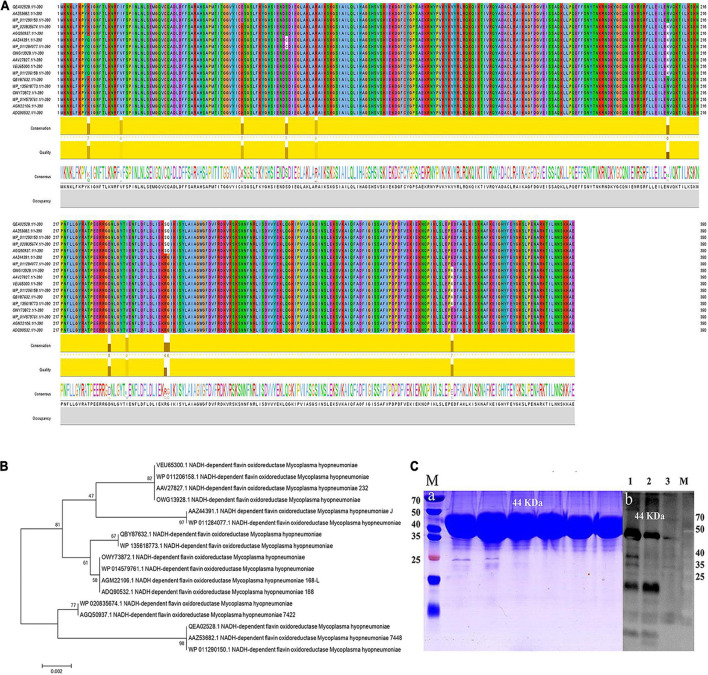
Multiple sequence alignment and evolutionary analyses of the NFOR protein sequences from various Mhp strains. **(A)** Sequence alignment of NFOR proteins among various Mhp strains using Clustal W by JavaView software. Sequences were derived from the UniProt database, and the consensus is shown with a web logo. **(B)** Phylogenetic analysis based on the NFOR proteins of different Mhp strains using the neighbor-joining (NJ) method with the sequences derived from the NCBI and UniProt databases. The number of phylogenetic branches is expressed as a bootstrap value (2,000 replicates) as a percentage of support for each group. The bars represent genetic distance. **(C)** Prokaryotic expression and purification of recombinant protein. WB verification of the prepared polyclonal antibody against Mhp NFOR. **(a)** Lane M: prestained protein mass markers, with recombinant rNFOR protein having a size of 44 kDa. **(b)** Lane 1, unpurified *E. coli* whole bacterial lysate containing rNFOR. Lanes 2 and 3, purified recombinant rNFOR protein at different loading concentrations (concentration of Lane 3 was half that of Lane 2).

Seventeen protein sequences of NFOR from Mhp strains were retrieved from the NCBI and UniProt databases, and the homologies between the strains were analyzed. As shown in Figure 2A, all the protein sequences were aligned with the CLUSTAL W program (Figure 2A). Molecular Evolutionary Genetics Analysis version 10 (MEGA 10) was used to make phylogenetic inferences based on the neighbor-joining criterion. The robustness of the hypothesis was tested through 2,000 non-parametric bootstrap analyses (Figure 2B). It was found that all the Mhp NFOR proteins had only minimal differences in amino acid positions according to the results of multiple sequence alignments. Similarly, evolutionary analysis revealed that the overall homology of NFOR among all the Mhp strains reached approximately 96.92%, suggesting that NFOR is a relatively highly conserved protein and exhibits few differences among different Mhp strains.

**FIGURE 3:**
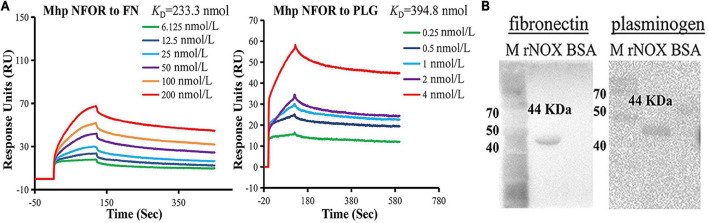
Identification of rNFOR binding ligands. **(A)** Mhp NFOR in gradient concentrations from 0.25 to 4 nmol/L and 6.125 to 200 nmol/L flowed through immobilized fibronectin and plasminogen in SPR assays. The concentrations of the proteins are consistent with the colored lines. RU, resonance units. **(B)** Mhp NFOR interaction with fibronectin and plasminogen as analyzed by Far-Western blotting. Lane M: prestained protein mass marker. Lane rNFOR: positive control, Mhp NFOR protein incubated with the anti-rNFOR antibody, fibronectin or plasminogen as well as the anti-fibronectin or anti-plasminogen antibody. Lane BSA: negative control, BSA incubated with the anti-rNFOR antibody, fibronectin or plasminogen as well as the anti-fibronectin or anti-plasminogen antibody.

The full-length coding sequence (CDS) of NFOR from Mhp 168 strain is 1,173 bp, with the predicted protein containing 390 amino acids. The signal peptide prediction results showed that there was no signal peptide because the probability was 0.137% as predicted by SignalP-5.0 Server, which indicates no possibility of signal peptide existence. On the basis of codon optimization, a prokaryotic expression vector pET-32a-NFOR was constructed, and the protein was expressed in *E. coli* BL21 (DE3). A total of 12 mg of recombinant rNFOR protein per liter of bacterial liquid was obtained after protein purification. The expression of the purified recombinant rNFOR protein from the pET-32a expression vector is shown in panel “a” of Figure 2C, with a recombinant rNFOR protein size of 44 kDa. The expression of rNFOR was detected using a polyclonal antibody in a Western blotting experiment. As shown in panel “b” of Figure 3B, a clear band appeared at position 44 kDa in both the whole bacterial protein sample and the purified rNFOR protein sample. After purification of the rNFOR protein, the enzymatic specific activity of the rNFOR protein was determined to be 29.12 IU/mg.

### Nicotinamide Adenine Dinucleotide-Dependent Flavin Oxidoreductase Is Located at the Surface of *Mycoplasma hyopneumoniae* Cells

**FIGURE 4:**
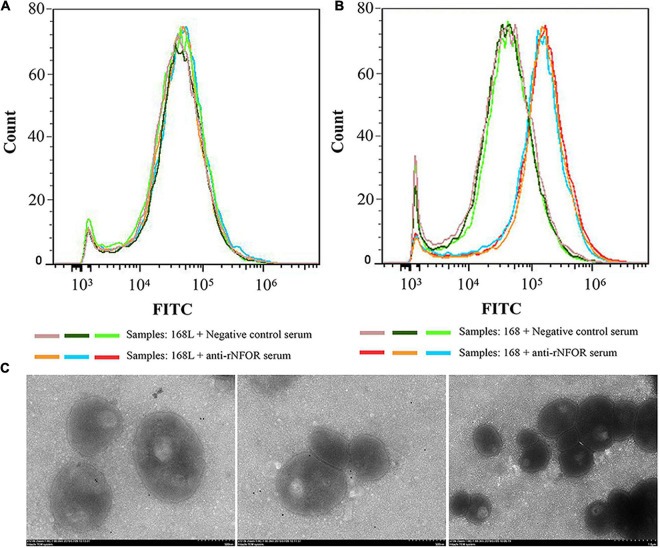
Detection of surface expression of NFOR by flow cytometry and immunoelectron microscopy techniques. Comparison of the fluorescence intensity of Mhp strains that differed in virulence treated with anti-NFOR serum and negative control serum. Fluorescence intensity was assessed by FITC staining with flow cytometry, 20,000 cells in three independent replicates were counted. **(A)** Mhp strain 168L treated with negative control preimmune serum or anti-rNFOR serum; **(B)** Mhp strain 168 treated with negative control preimmune serum or anti-rNFOR serum; **(C)** From left to right are the Mhp strains JS and 168 treated with anti-Mhp NFOR and preimmune serum before treatment with secondary gold-conjugated antibodies, respectively.

To confirm whether NFOR is located on the surface of Mhp, two tests were performed. Flow cytometry analysis revealed that the outer membrane-localized NFOR can be recognized by the NFOR-specific antibody on the surface of both the 168 and 168L Mhp strains, indicating that the NFOR antigen is present on the cell surface of Mhp. There was no significant difference in the mean fluorescence intensity (MFI) between Mhp strain 168L incubated with preimmune serum and Mhp strain 168L treated with anti-recombinant protein NFOR (anti-rNFOR) serum (Figure 4A), while the MFI of strain 168 treated with preimmune serum was approximately 4-fold lower than that of Mhp 168 treated with anti-rNFOR serum (Figure 4B). In addition, antibodies against the Mhp NFOR protein bound to the surface of Mhp cells, as revealed by immune electron microscopy. The antibodies were localized and restricted to the peripheral area of Mhp cells in both the Mhp strain JS and strain 168, while in the preimmune serum-treated negative group, gold particles were not visible (Figure 4C).

### rNFOR Adheres to Immortalized Porcine Bronchial Epithelial Cells, and Treatment With Anti-rNFOR Serum Inhibits Adherence to *Mycoplasma hyopneumoniae*

**FIGURE 5:**
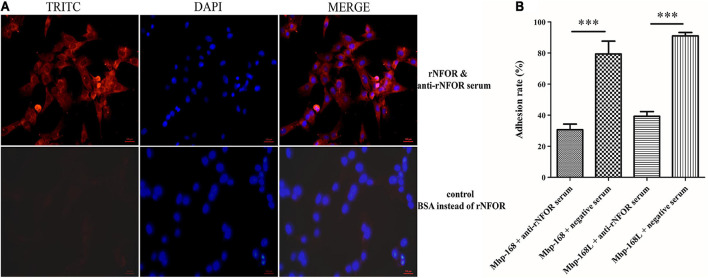
rNFOR adhesion and adhesion inhibition assays using IFA and real-time PCR analysis. **(A)** hTERT-PBECs were fixed and incubated with purified rNFOR or BSA (control) and anti-rNFOR rabbit polyclonal antibody before incubation with the TRITC-conjugated anti-IgG fluorescent antibody. TRITC: An anti-rabbit IgG antibody (orange-red) conjugated to TRITC was used to observe the reactivity of the antibody to IgG; DAPI: 4′,6-diamidino-2-phenylindole, the nuclei of all cells were stained with DAPI Reagent (blue); the merged images are shown in the column labeled “merged”. The images were captured at 400× magnification. The red bar represents the scale: 100 μm. **(B)** Mhp strains 168 and 168L were preincubated with polyclonal antibodies against rNFOR or preimmune serum before inoculation with hTERT-PBECs. The adhesion rate was calculated by real-time PCR to count Mhp bacteria. Adhesion rate = (number of Mhp antigens harvested from infected cells incubated with anti-rNOX serum/number of Mhp antigens harvested from infected cells incubated with preimmune serum) × 100. Quantitative real-time PCR analysis was performed, and the data are expressed as log10 DNA copy number per mL from cell distribution against P97 infected with Mhp. The data are presented as the mean ± SD from at least three independent replicates (^∗∗∗^ means extremely significant differences, with *p* < 0.001).

An indirect immunofluorescence assay (IFA) was used to determine whether Mhp NFOR could specifically adhere to the surface of hTERT-PBECs. The results revealed orange-red punctate fluorescence on the surface of immortalized hTERT-PBECs incubated with rNFOR, whereas in the negative control group, no specific fluorescence was observed around the cell nuclei stained with DAPI (Figure 5A), suggesting that rNFOR could specifically bind to hTERT-PBECs.

To further assess the role of NFOR in Mhp adhesion to hTERT-PBECs, an antibody inhibition assay was conducted. Anti-rNFOR serum decreased the adherence of Mhp (both strains 168 and 168L) to hTERT-PBECs compared to that of the control group, which was treated with negative serum. The adhesion level was shown as the adhesion rate compared with the adhesion rate of Mhp in the absence of the anti-rNFOR polyclonal antibody. Incubation with the anti-rNFOR antibody led to 48.7% (strain 168) and 51.75% (strain 168L) (*P* < 0.001) reductions in the adhesion rate of Mhp to hTERT-PBECs (Figure 5B). The results further indicated that NFOR of Mhp plays an irreplaceable role in the adherence of Mhp to host cells.

### Identification of rNFOR Binding Ligands

To determine whether components of hTERT-PBECs interact with NFOR, SPR, and far-Western blotting (Far-WB) analysis were used to examine the interactions between Mhp NFOR and fibronectin and plasminogen. Mhp NFOR could bind to fibronectin and plasminogen in a dose-dependent manner, with equilibrium dissociation constant (KD) values of 233.3 and 394.8 nmol, respectively (Figure 3A); these results indicated that Mhp NFOR could specifically bind to both fibronectin and plasminogen and had a relatively high affinity for fibronectin.

As shown in Figure 3B, before the final reaction with fibronectin or plasminogen, a corresponding band was observed in the reaction of rNFOR with the anti-rNFOR antibody (positive control), but no specific reaction was observed in the negative control (BSA was used instead of anti-rNFOR antibody). The results showed that Mhp NFOR has a strong affinity for plasminogen and fibronectin and that the binding ability of NFOR and fibrinogen is better than that of Mhp and plasminogen.

### rNFOR Induces Cytotoxicity, Oxidative Stress Damage, and Apoptosis of Immortalized Porcine Bronchial Epithelial Cells

**FIGURE 6:**
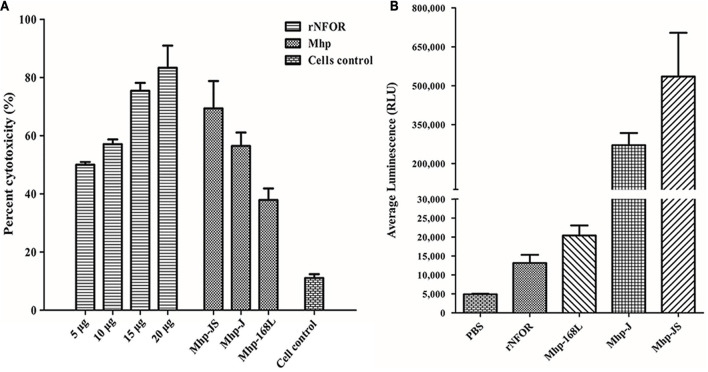
Cytotoxicity and oxidative stress of hTERT-PBECs induced by rNFOR. **(A)** A CytoTox 96^®^ Non-Radioactive Cytotoxicity Assay was performed after treatment with different concentrations of rNFOR and Mhp strains with different virulences (positive control, JS, J, and 168L). **(B)** ROS-Glo^TM^ H_2_O_2_ assay of cellular oxidative stress after treatment with rNFOR and Mhp strains with different virulences (JS, J, and 168L).

The cytotoxic effects of rNFOR on hTERT-PBECs were determined by the CytoTox 96^®^ Non-Radioactive Cytotoxicity test, and it was found that the cell viability was significantly reduced by rNFOR in a dose-dependent manner (Figure 6A). In addition, rNFOR induced oxidative stress damage in hTERT-PBECs (Figure 6B). The results also demonstrated that the cytotoxic and host cell oxidative stress-inducing effects of Mhp against hTERT-PBECs were significantly correlated with the different degrees of virulence of the pathogenic Mhp strains (Figures 6A,B).

**FIGURE 7:**
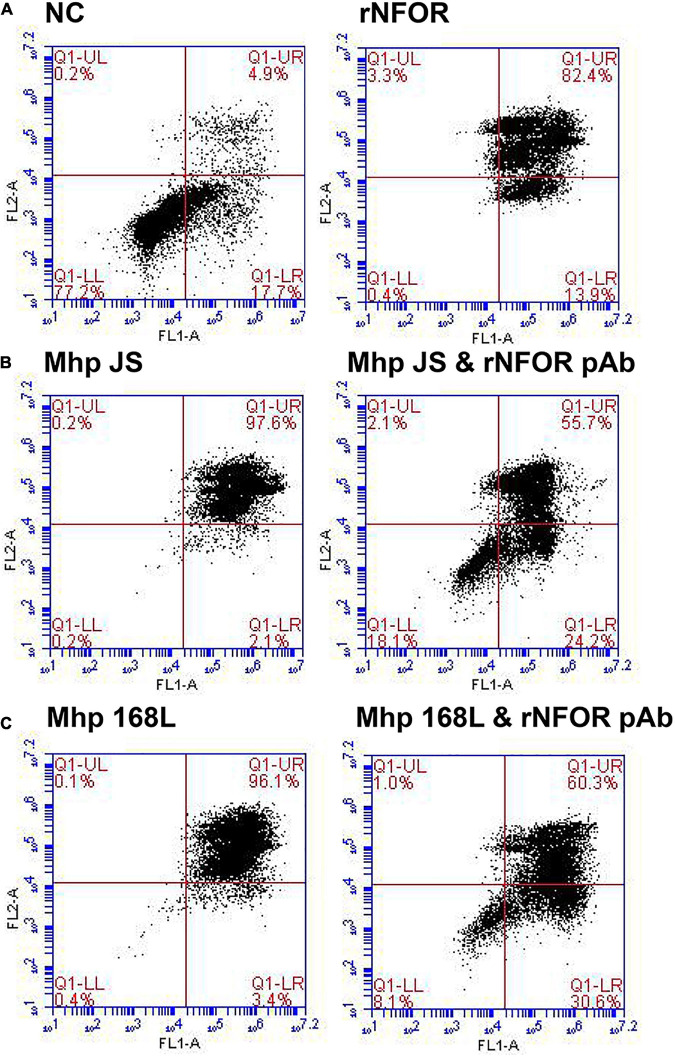
Apoptosis of hTERT-PBECs induced by rNFOR and the Mhp strains JS and 168L as determined by the annexin V FITC/PI double staining method 12 h post infection. The late apoptosis rate (upper right quadrant) showed a substantial difference. **(A)** Negative control hTERT-PBECs cultured for 12 h after reaching 80% confluence in DMEM/F12 without FBS and growth factors; rNFOR indicates the apoptosis rate of hTERT-PBECs treated with recombinant rNOX protein. **(B)** hTERT-PBECs incubated with the Mhp high-virulence strain JS for 12 h treated without and with the rNFOR polyclonal antibody. **(C)** hTERT-PBECs incubated with the Mhp low-virulence strain 168L for 12 h treated without and with the rNFOR polyclonal antibody.

The flow cytometry results further indicated that rNFOR could induce apoptosis in hTERT-PBECs (Figure 7A), and the late-stage apoptosis (upper right quadrant) rate was increased by 82.4% compared with the negative control group (4.9%). The late-stage apoptosis induced by the Mhp strain JS or 168L in hTERT-PTECs was significantly decreased when rNFOR was blocked with an anti-rNFOR antibody. The late-stage apoptotic cell percentage decreased by 41.9% and 35.8%, respectively (Figures 7B,C).

## Discussion

To date, all mycoplasmas cultured and identified are known to be parasites, and the hosts of these host include humans or animals, with a higher degree of specificity to hosts and tissues (Gautier-Bouchardon, 2018). The main habitats of mycoplasmas are the respiratory and genitourinary tracts, the serous membranes and the epithelial surfaces of mammary glands of certain animal species. At present, >90 bacterial species have one or more families of proteins that function outside of their typical roles to aid colonization and induce disease. As Mycoplasma have reduced genomes, these off-target effects might be evolutionarily favored. Mhp is a host-specific pathogen that infects only pigs. Currently, many virulence factors of Mhp have been identified, and their roles include adhesion, invasion, and intracellular proliferation; however, the molecular mechanisms underlying infection and pathogenesis have not been fully elucidated (Maes et al., 2018). In recent years, some studies have indicated that several metabolic enzymes, including enolase and EF-TU of Mhp, which were investigated by our team (Hegde et al., 2015; Yu et al., 2018; Chen et al., 2019), play an important role in host-pathogen interactions during *Mycoplasma* infections; these findings indicate that the two metabolic enzymes act as virulence proteins in addition to their typical roles.

“Omics” studies have shown that there are significant differences in the expression of NFOR between the high-virulence Mhp strain 7,448 and the moderate-virulence strain J and nonpathogenic *M. flocculare* through transcriptomics comparison (Siqueira et al., 2014) or between the high- and low-virulence Mhp strains through proteomics comparison (Reolon et al., 2014; Li et al., 2019); however, a supplementary file of a previous study classified NFOR as a “nonvirulent” protein (Li et al., 2019), but no verification was provided. However, here, the mRNA expression levels of the NFOR gene in the pathogenic high-virulence Mhp strains 168, LH, and JS were significantly upregulated compared with those in either the moderate-virulence strain J or low-virulence strain 168L. This result preliminarily confirmed that expression of NFOR was correlated with the virulence of Mhp. Further investigation showed that the NFOR antigen was located on the cell surface of Mhp by both flow cytometric MFI analysis and immunoelectron microscopy observation. Compared with the nonpathogenic 168L strain, the fluorescence intensity of the pathogenic high-virulence strain 168 was increased by approximately 4-fold, further indicating that NFOR may be responsible for the virulence of Mhp.

Several dozen proteins were previously identified to be ubiquitous intracellular enzymes or intracellular/surface moonlighting proteins (ISMPs) that have a canonical function in essential cellular processes (Jeffery, 2018). Therefore, a major question arises, that is, similar to most ISMPs, how does NFOR secrete, become attached to the cell surface, and then perform an extracytoplasmic function. Several studies have proposed some hypotheses. For example, purified proteins have shown that some ISMPs can adhere to the cell surface by reassociation in both Gram-positive and Gram-negative bacteria; therefore, it is possible that some ISMPs are secreted and then reassociate with the cell surface after secretion. An increase in extracellular pH has been shown to cause some *Lactobacillus crispatus* ISMPs to be released from the cell surface (Antikainen et al., 2007). In most cases, it is unknown which components of the cell surface (for example, proteins and lipids, etc.) bind to ISMPs. However, recent studies have shown that extracellular enolase is bound to a rhamnose residue in the cell membrane of mycoplasma (Daubenspeck et al., 2016), and enolase and GAPDH can covalently bind to the lipotectoic acid on *L. crispatus* (Antikainen et al., 2007). Although we still do not know what mechanism NFOR uses as an enzyme in the cell matrix to act as a potential adhesin on the cell surface, the investigations described above may provide some inspiration for our further study.

The adhesion of all microorganisms, including mycoplasma, to their host cells is a key step for their colonization and subsequent infection of the host. Adhesion ability is an important factor that reflects bacterial virulence (Zhao et al., 2017). Mhp is mainly found on the mucosal surface along the entire swine respiratory tract, including the trachea, bronchi and bronchioles, inducing ciliostasis and loss of cilia (Blanchard et al., 1992). The first stage of pathogenesis is the adhesion of Mhp to the cilia of the epithelial cells of the respiratory mucosa by means of adhesins. Here, we found that NFOR could adhere to the hTERT-PBECs established in our previous study (Xie et al., 2018). In addition, preneutralizing Mhp with polyclonal antiserum to rNFOR obviously decreased the adherence of Mhp to host cells.

Fibronectin has been studied more extensively, and it has been identified as a popular extracellular matrix protein that forms a molecular bridge between pathogens and host cell receptors (Schwarz-Linek et al., 2006; Henderson et al., 2011). According to our previous studies (Yu et al., 2018; Chen et al., 2019), several fibronectin-binding bacterial proteins have been found to mediate the adhesion of bacteria to host cells and subsequent invasion by binding to fibronectin. Therefore, fibronectin binding protein plays a critical role in the pathogenic process of bacteria, including mycoplasmas. In addition to fibronectin, a variety of bacteria can sequester the host zymogen plasminogen to the cell surface. Once localized to the bacterial surface, plasminogen can act as a cofactor for adhesion or, after being activated as plasmin, can provide an effective source of proteolytic activity. The recruitment of plasminogen to the surface of bacterial cells is directly mediated by specialized cell surface receptors or cytoplasmic and glycolytic pathway proteins located on the surface of bacterial cells or is indirectly mediated through interactions with host plasma proteins such as fibrinogen (Sanderson-Smith et al., 2012). In this study, we found that rNFOR could specifically bind to fibronectin and plasminogen and had a higher affinity for fibronectin. It is possible that fibronectin might function as the main receptor of NFOR and that plasminogen acts as a cofactor to mediate the adhesion process of Mhp. Further characterization of the interaction between fibronectin and plasminogen would be useful and helpful for improving the understanding of the adhesion-related factors of Mhp.

Lactate dehydrogenase is a stable cytoplasmic enzyme that converts lactate to pyruvate. Analyses of LDH release by damaged cells are one method used to detect cell viability from the perspective of cell membrane integrity (Jing et al., 2015). Our results showed that NFOR could induce cytotoxicity in hTERT-PBECs in a dose-dependent manner, and the NFOR-triggered LDH release by host cells was positively correlated with the different virulences of the various Mhp strains. ROS are constantly generated during numerous cellular processes, one of the main sources of which is aerobic respiration, but most are often offset by antioxidant proteins. In addition, a large amount of ROS is produced from bacterial or viral infections, inflammatory reactions, ionizing radiation, and many chemical drugs (Bai et al., 2013; Grant and Hung, 2013; Zhang et al., 2019). In these processes, if the induced levels of ROS exceed the upper limit offset by antioxidant proteins, so-called “oxidative stress” will be generated. Unlike cell necrosis, apoptosis is not a passive process but a phenomenon that induces autologous injury through a series of signal activation, protein expression and regulation processes (Obeng, 2021). A recent study demonstrated that ROS deposition is thought to be a direct cause of apoptosis because ROS induce strong cytotoxicity to host cells. Thus, when the intracellular ROS content substantially increases, it stimulates oxidative stress and induces apoptosis (Simon et al., 2000; Halliwell and Whiteman, 2004; Li et al., 2016).

In this study, we found that rNFOR from Mhp strains that differed in virulence could induce oxidative stress damage in hTERT-PBECs. Moreover, the release of H_2_O_2_ from cells induced by the low-virulence Mhp strain 168L was significantly lower than that induced by a high-virulence strain and a moderate-virulence strain (JS and J). Flow cytometry results further indicated that rNFOR could also induce apoptosis in hTERT-PBECs, and the late-stage apoptosis rate was increased by 82.4% compared with the negative control (4.9%) after changing the medium to maintenance medium without FBS and growth factors after 12 h. The late-stage apoptosis induced by either the high-virulence Mhp strain JS or the low-virulence Mhp strain 168L in hTERT-PBECs was significantly decreased when rNFOR was blocked with an anti-rNFOR polyclonal antibody, with late-stage apoptotic cell percentages decreasing by 41.9 and 35.8%, respectively. In conclusion, these results suggested that NFOR, functioning as more than only a metabolic enzyme, may act as a potential novel virulence factor of Mhp, and these findings will provide certain theoretical support and new ideas for the research and development of live-attenuated or subunit vaccines against Mhp.

## Data Availability Statement

The original contributions presented in the study are included in the article/supplementary material, further inquiries can bedirected to the corresponding authors. Moreover, genome sequencing information of Mhp LH has been submitted to NCBI, with the GenBank accession number CP079799.

## Ethics Statement

The animal study was reviewed and approved by the Committee on the Ethics of Animal Experiments and performed in Jiangsu Academy of Agricultural Sciences, with the approval numbers SYXK (Su) 2015-0019 and SYXK (Su)2020-0023. Animal experiments, including sample collection, were conducted in strict accordance with the guidelines of Jiangsu Province Animal Regulations (Government Decree No. 45).

## Author Contributions

XX carried out most of the experiments described in the manuscript and wrote the manuscript. FH, RC, JW, and JL helped to prepare the recombinant protein and the rabbit hyperimmune sera. YW cultured the Mhp strains. HW and ZZ helped to perform the relevant *in vivo* cell cytotoxicity and cell apoptosis experiments. YB performed the real-time PCR experiments. QX and GS helped to revise the manuscript. ZF conceived the study and contributed in its design and coordination. All authors read and approved the final manuscript.

## Conflict of Interest

The authors declare that the research was conducted in the absence of any commercial or financial relationships that could be construed as a potential conflict of interest.

## Publisher’s Note

All claims expressed in this article are solely those of the authors and do not necessarily represent those of their affiliated organizations, or those of the publisher, the editors and the reviewers. Any product that may be evaluated in this article, or claim that may be made by its manufacturer, is not guaranteed or endorsed by the publisher.

## Footnotes

1
http://www.cbs.dtu.dk/services/SignalP/

## References

[B1] Almagro Armenteros J. J. Tsirigos K. D. Sonderby C. K. Petersen T. N. Winther O. Brunak S. (2019). SignalP 5.0 improves signal peptide predictions using deep neural networks. *Nat. Biotechnol.* 37 420–423. 10.1038/s41587-019-0036-z 30778233

[B2] Antikainen J. Kuparinen V. Lahteenmaki K. Korhonen T. K. (2007). pH-dependent association of enolase and glyceraldehyde-3-phosphate dehydrogenase of *Lactobacillus crispatus* with the cell wall and lipoteichoic acids. *J. Bacteriol.* 189 4539–4543. 10.1128/jb.00378-07 17449624 PMC1913374

[B3] Bai F. Ni B. Liu M. Feng Z. Xiong Q. Xiao S. (2013). *Mycoplasma hyopneumoniae*-derived lipid-associated membrane proteins induce apoptosis in porcine alveolar macrophage via increasing nitric oxide production, oxidative stress, and caspase-3 activation. *Vet. Immunol. Immunopathol.* 155 155–161. 10.1016/j.vetimm.2013.07.004 23928261

[B4] Blanchard B. Vena M. M. Cavalier A. Le Lannic J. Gouranton J. Kobisch M. (1992). Electron microscopic observation of the respiratory tract of SPF piglets inoculated with *Mycoplasma hyopneumoniae*. *Vet. Microbiol.* 30 329–341. 10.1016/0378-1135(92)90020-t 1533978

[B5] Buim M. R. Buzinhani M. Yamaguti M. Oliveira R. C. Mettifogo E. Ueno P. M. (2011). *Mycoplasma synoviae* cell invasion: elucidation of the *Mycoplasma* pathogenesis in chicken. *Comp. Immunol. Microbiol. Infect. Dis.* 34 41–47. 10.1016/j.cimid.2009.11.001 19969353

[B6] Burki S. Gaschen V. Stoffel M. H. Stojiljkovic A. Frey J. Kuehni-Boghenbor K. (2015). Invasion and persistence of *Mycoplasma bovis* in embryonic calf turbinate cells. *Vet. Res.* 46:53. 25976415 10.1186/s13567-015-0194-z PMC4432498

[B7] Chen R. Yu Y. Feng Z. Gan R. Xie X. Zhang Z. (2019). Featured species-specific loops are found in the crystal structure of Mhp Eno, a cell surface adhesin from *Mycoplasma hyopneumoniae*. *Front. Cell. Infect. Microbiol.* 9:209. 10.3389/fcimb.2019.00209 31263685 PMC6585157

[B8] Chopra-Dewasthaly R. Baumgartner M. Gamper E. Innerebner C. Zimmermann M. Schilcher F. (2012). Role of Vpma phase variation in *Mycoplasma agalactiae* pathogenesis. *FEMS Immunol. Med. Microbiol.* 66 307–322. 22809092 10.1111/j.1574-695X.2012.01010.x PMC4510919

[B9] Daubenspeck J. M. Liu R. Dybvig K. (2016). Rhamnose links moonlighting proteins to membrane phospholipid in mycoplasmas. *PLoS One* 11:e0162505. 10.1371/journal.pone.0162505 27603308 PMC5014317

[B10] Furr P. M. Taylor-Robinson D. (1993). Factors influencing the ability of different mycoplasmas to colonize the genital tract of hormone-treated female mice. *Int. J. Exp. Pathol.* 74 97–101. 8471540 PMC2002218

[B11] Gautier-Bouchardon A. V. (2018). Antimicrobial resistance in *Mycoplasma* spp. *Microbiol. Spectr.* 6 1–21. 10.1128/microbiolspec.arba-0030-2018 PMC11633602 30003864

[B12] Grant S. S. Hung D. T. (2013). Persistent bacterial infections, antibiotic tolerance, and the oxidative stress response. *Virulence* 4 273–283. 10.4161/viru.23987 23563389 PMC3710330

[B13] Halliwell B. Whiteman M. (2004). Measuring reactive species and oxidative damage in vivo and in cell culture: how should you do it and what do the results mean? *Br. J. Pharmacol.* 142 231–255. 10.1038/sj.bjp.0705776 15155533 PMC1574951

[B14] Hegde S. Hegde S. Spergser J. Brunthaler R. Rosengarten R. Chopra-Dewasthaly R. (2014). *In vitro* and *in vivo* cell invasion and systemic spreading of *Mycoplasma agalactiae* in the sheep infection model. *Int. J. Med. Microbiol. IJMM* 304 1024–1031. 25129554 10.1016/j.ijmm.2014.07.011 PMC4282308

[B15] Hegde S. Rosengarten R. Chopra-Dewasthaly R. (2015). Disruption of the pdhB pyruvate dehydrogenase [corrected] gene affects colony morphology, *in vitro* growth and cell invasiveness of *Mycoplasma agalactiae*. *PloS One* 10:e0119706. 10.1371/journal.pone.011970 25799063 PMC4370745

[B16] Hegermann J. Halbedel S. Dumke R. Regula J. Gabdoulline R. R. Mayer F. (2008). The acidic, glutamine-rich Mpn474 protein of *Mycoplasma pneumoniae* is surface exposed and covers the complete cell. *Microbiology* 154 1185–1192. 10.1099/mic.0.2007/013342-0 18375811

[B17] Henderson B. (2014). An overview of protein moonlighting in bacterial infection. *Biochem. Soc. Trans.* 42 1720–1727. 10.1042/bst20140236 25399596

[B18] Henderson B. Nair S. Pallas J. Williams M. A. (2011). Fibronectin: a multidomain host adhesin targeted by bacterial fibronectin-binding proteins. *FEMS Microbiol. Rev.* 35 147–200. 10.1111/j.1574-6976.2010.00243.x 20695902

[B19] Ho C. Chu T. Chin H. Mao H. Yeh A. Chen C. (1980). Microagglutination test for the diagnosis of swine mycoplasmal pneumonia and the identification of Mycoplasmas. *Acta Vet. Zootech. Sinica* 11 175–186.

[B20] Jeffery C. (2018). Intracellular proteins moonlighting as bacterial adhesion factors. *AIMS Microbiol.* 4 362–376. 10.3934/microbiol.2018.2.362 31294221 PMC6604927

[B21] Jing X. Park J. H. Peters T. M. Thorne P. S. (2015). Toxicity of copper oxide nanoparticles in lung epithelial cells exposed at the air-liquid interface compared with *in vivo* assessment. *Toxicol. In Vitro* 29 502–511. 10.1016/j.tiv.2014.12.023 25575782 PMC4373347

[B22] Kumar S. Stecher G. Li M. Knyaz C. Tamura K. (2018). MEGA X: molecular evolutionary genetics analysis across computing platforms. *Mol. Biol. Evol.* 35 1547–1549. 10.1093/molbev/msy096 29722887 PMC5967553

[B23] Larkin M. A. Blackshields G. Brown N. P. Chenna R. Mcgettigan P. A. Mcwilliam H. (2007). Clustal W and Clustal X version 2.0. *Bioinformatics* 23 2947–2948. 10.1093/bioinformatics/btm404 17846036

[B24] Leal Zimmer F. M. A. Paes J. A. Zaha A. Ferreira H. B. (2020). Pathogenicity & virulence of *Mycoplasma hyopneumoniae*. *Virulence* 11 1600–1622. 33289597 10.1080/21505594.2020.1842659 PMC7733983

[B25] Leigh S. A. Evans J. D. Branton S. L. Collier S. D. (2008). The effects of increasing sodium chloride concentration on *Mycoplasma gallisepticum* vaccine survival in solution. *Avian Dis.* 52 136–138. 10.1637/7979-040507-resnote 18459310

[B26] Li S. Fang L. Liu W. Song T. Zhao F. Zhang R. (2019). Quantitative proteomic analyses of a pathogenic strain and its highly passaged attenuated strain of *Mycoplasma hyopneumoniae*. *Biomed. Res. Int.* 2019:4165735. 31355261 10.1155/2019/4165735 PMC6634062

[B27] Li Y. Jiang Z. Xue D. Deng G. Li M. Liu X. (2016). *Mycoplasma ovipneumoniae* induces sheep airway epithelial cell apoptosis through an ERK signalling-mediated mitochondria pathway. *BMC Microbiol.* 16:222. 10.1186/s12866-016-0842-0 27663303 PMC5035462

[B28] Liu J. Yin Y. Song Z. Li Y. Jiang S. Shao C. (2014). NADH: flavin oxidoreductase/NADH oxidase and ROS regulate microsclerotium development in *Nomuraea rileyi*. *World J. Microbiol. Biotechnol.* 30 1927–1935. 10.1007/s11274-014-1610-7 24497186

[B29] Liu W. Xiao S. Li M. Guo S. Li S. Luo R. (2013). Comparative genomic analyses of *Mycoplasma hyopneumoniae* pathogenic 168 strain and its high-passaged attenuated strain. *BMC Genomics* 14:80. 10.1186/1471-2164-14-80 23384176 PMC3626624

[B30] Maes D. Sibila M. Kuhnert P. Segales J. Haesebrouck F. Pieters M. (2018). Update on *Mycoplasma hyopneumoniae* infections in pigs: knowledge gaps for improved disease control. *Transbound. Emerg. Dis.* 65(Suppl. 1) 110–124. 10.1111/tbed.12677 28834294

[B31] Montfort-Gardeazabal J. M. Balderas-Renteria I. Casillas-Vega N. G. Zarate X. (2021). Expression and purification of the antimicrobial peptide Bin1b in *Escherichia coli* tagged with the fusion proteins CusF3H+ and SmbP. *Protein Expr. Purif.* 178:105784. 10.1016/j.pep.2020.105784 33129981

[B32] Much P. Winner F. Stipkovits L. Rosengarten R. Citti C. (2002). *Mycoplasma gallisepticum*: influence of cell invasiveness on the outcome of experimental infection in chickens. *FEMS Immunol. Med. Microbiol.* 34 181–186. 10.1111/j.1574-695x.2002.tb00622.x 12423769

[B33] Obeng E. (2021). Apoptosis (programmed cell death) and its signals–a review. *Braz. J. Biol.* 81 1133–1143. 10.1590/1519-6984.228437 33111928

[B34] Peterson S. N. Fraser C. M. (2001). The complexity of simplicity. *Genome Biol.* 2:Comment2002.1. 11182883 10.1186/gb-2001-2-2-comment2002 PMC138898

[B35] Pilo P. Vilei E. M. Peterhans E. Bonvin-Klotz L. Stoffel M. H. Dobbelaere D. (2005). A metabolic enzyme as a primary virulence factor of *Mycoplasma mycoides* subsp. mycoides small colony. *J. Bacteriol.* 187 6824–6831. 10.1128/jb.187.19.6824-6831.2005 16166545 PMC1251598

[B36] Razin S. Yogev D. Naot Y. (1998). Molecular biology and pathogenicity of mycoplasmas. *Microbiol. Mol. Biol. Rev. MMBR* 62 1094–1156. 9841667 10.1128/mmbr.62.4.1094-1156.1998 PMC98941

[B37] Reolon L. A. Martello C. L. Schrank I. S. Ferreira H. B. (2014). Survey of surface proteins from the pathogenic *Mycoplasma hyopneumoniae* strain 7448 using a biotin cell surface labeling approach. *PloS One* 9:e112596. 10.1371/journal.pone.0112596 25386928 PMC4227723

[B38] Rohmer L. Hocquet D. Miller S. I. (2011). Are pathogenic bacteria just looking for food? Metabolism and microbial pathogenesis. *Trends Microbiol.* 19 341–348. 10.1016/j.tim.2011.04.003 21600774 PMC3130110

[B39] Sanderson-Smith M. L. De Oliveira D. M. Ranson M. McArthur J. D. (2012). Bacterial plasminogen receptors: mediators of a multifaceted relationship. *J. Biomed. Biotechnol.* 2012:272148. 23118502 10.1155/2012/272148 PMC3478875

[B40] Schwarz-Linek U. Hook M. Potts J. R. (2006). Fibronectin-binding proteins of Gram-positive cocci. *Microbes Infect.* 8 2291–2298. 10.1016/j.micinf.2006.03.011 16782385

[B41] Simon H. U. Haj-Yehia A. Levi-Schaffer F. (2000). Role of reactive oxygen species (ROS) in apoptosis induction. *Apoptosis* 5 415–418. 11256882 10.1023/a:1009616228304

[B42] Siqueira F. M. Gerber A. L. Guedes R. L. Almeida L. G. Schrank I. S. Vasconcelos A. T. (2014). Unravelling the transcriptome profile of the swine respiratory tract mycoplasmas. *PloS one* 9:e110327. 10.1371/journal.pone.0110327 25333523 PMC4198240

[B43] Wu Y. Jin M. Bai F. Zhang X. Hua L. Lei Z. (2012). Development and application of TaqMan-BHQ real time PCR assay for detection of *Mycoplasma hyopneumoniae* P97. *Chin. Vet. Sci.* 42 1268–1272.

[B44] Xie X. Gan Y. Pang M. Shao G. Zhang L. Liu B. (2018). Establishment and characterization of a telomerase-immortalized porcine bronchial epithelial cell line. *J. Cell. Physiol.* 233 9763–9776. 10.1002/jcp.26942 30078190

[B45] Xie X. Pang M. Liang S. Lin Y. Zhao Y. Qiu D. (2021). Cellular microRNAs influence replication of H3N2 canine influenza virus in infected cells. *Vet. Microbiol.* 257:109083. 10.1016/j.vetmic.2021.109083 33894663

[B46] Xiong Q. Wei Y. Feng Z. Gan Y. Liu Z. Liu M. (2014). Protective efficacy of a live attenuated *Mycoplasma hyopneumoniae* vaccine with an ISCOM-matrix adjuvant in pigs. *Vet. J.* 199 268–274. 10.1016/j.tvjl.2013.11.001 24314715

[B47] Yu Y. Liu M. Hua L. Qiu M. Zhang W. Wei Y. (2018). Fructose-1,6-bisphosphate aldolase encoded by a core gene of *Mycoplasma hyopneumoniae* contributes to host cell adhesion. *Vet. Res.* 49:114. 30454073 10.1186/s13567-018-0610-2 PMC6245935

[B48] Zhang Z. Rong L. Li Y. P. (2019). Flaviviridae viruses and oxidative stress: implications for viral pathogenesis. *Oxid. Med. Cell. Longev.* 2019:1409582. 31531178 10.1155/2019/1409582 PMC6720866

[B49] Zhao G. Zhang H. Chen X. Zhu X. Guo Y. He C. (2017). *Mycoplasma bovis* NADH oxidase functions as both a NADH oxidizing and O2 reducing enzyme and an adhesin. *Sci. Rep.* 7:44. 28246386 10.1038/s41598-017-00121-y PMC5427908

[B50] Zhu W. Zhang Q. Li J. Wei Y. Cai C. Liu L. (2017). Glyceraldehyde-3-phosphate dehydrogenase acts as an adhesin in *Erysipelothrix rhusiopathiae* adhesion to porcine endothelial cells and as a receptor in recruitment of host fibronectin and plasminogen. *Vet. Res.* 48:16. 28327178 10.1186/s13567-017-0421-x PMC5360030

